# Bias in estimates of variance components in populations undergoing genomic selection: a simulation study

**DOI:** 10.1186/s12864-019-6323-8

**Published:** 2019-12-09

**Authors:** Hongding Gao, Per Madsen, Gert Pedersen Aamand, Jørn Rind Thomasen, Anders Christian Sørensen, Just Jensen

**Affiliations:** 1 Center for Quantitative Genetics and Genomics, Department of Molecular Biology and Genetics, Aarhus University, DK-8830 Tjele, Denmark; 2 Nordic Cattle Genetic Evaluation, DK-8200 Aarhus, Denmark; 3 VikingGenetics, DK-8960 Assentoft, Denmark

**Keywords:** Single-step, Imputation error, Genetic variance, Marker variance, Bayesian regression

## Abstract

**Background:**

After the extensive implementation of genomic selection (GS), the choice of the statistical model and data used to estimate variance components (VCs) remains unclear. A primary concern is that VCs estimated from a traditional pedigree-based animal model (P-AM) will be biased due to ignoring the impact of GS. The objectives of this study were to examine the effects of GS on estimates of VC in the analysis of different sets of phenotypes and to investigate VC estimation using different methods. Data were simulated to resemble the Danish Jersey population. The simulation included three phases: (1) a historical phase; (2) 20 years of conventional breeding; and (3) 15 years of GS. The three scenarios based on different sets of phenotypes for VC estimation were as follows: (1) Pheno_1_: phenotypes from only the conventional phase (1–20 years); (2) Pheno_1 + 2_: phenotypes from both the conventional phase and GS phase (1–35 years); (3) Pheno_2_: phenotypes from only the GS phase (21–35 years). Single-step genomic BLUP (ssGBLUP), a single-step Bayesian regression model (ssBR), and P-AM were applied. Two base populations were defined: the first was the founder population referred to by the pedigree-based relationship (P-base); the second was the base population referred to by the current genotyped population (G-base).

**Results:**

In general, both the ssGBLUP and ssBR models with all the phenotypic and genotypic information (Pheno_1 + 2_) yielded biased estimates of additive genetic variance compared to the P-base model. When the phenotypes from the conventional breeding phase were excluded (Pheno_2_), P-AM led to underestimation of the genetic variance of P-base. Compared to the VCs of G-base, when phenotypes from the conventional breeding phase (Pheno_2_) were ignored, the ssBR model yielded unbiased estimates of the total genetic variance and marker-based genetic variance, whereas the residual variance was overestimated.

**Conclusions:**

The results show that neither of the single-step models (ssGBLUP and ssBR) can precisely estimate the VCs for populations undergoing GS. Overall, the best solution for obtaining unbiased estimates of VCs is to use P-AM with phenotypes from the conventional phase or phenotypes from both the conventional and GS phases.

## Background

In animal breeding, the prediction of breeding values requires accurate and unbiased estimates of variance components (VCs). There are several approaches available for VC estimation depending on the practical conditions (for instance, population structure) [1]. Among these methods, restricted maximum likelihood (REML) [2] is a well-known method that has been widely applied under animal models to obtain genetic parameters for traits of interest [3–8]. Alternatively, Bayesian methods with Markov chain Monte-Carlo (MCMC) procedures such as Gibbs sampling can be adopted to obtain marginal inferences for VCs [9–12].

Before genomic information was available, Sorensen and Kennedy [13] showed that unbiased estimation of VCs in a base population could be obtained by an animal model when all data leading to selection decisions and complete relationships were incorporated, while ignoring data from selected ancestors led to biased estimation due to not accounting for gametic disequilibrium. Moreover, in small populations with few generations, an animal model with pedigree information can account for bias due to inbreeding and the Bulmer effect [14]. However, these results were obtained assuming the infinitesimal model. To date, although there are currently several genomic prediction models available, the choice of statistical model and which data should be used to estimate VCs in the genomics era remain unclear.

The use of genomic selection (GS) has greatly enhanced genetic gains due to improved selection accuracy and shortened generation intervals (e.g., dairy cattle breeding); this has led to faster changes in gene frequencies compared to traditional phenotype and pedigree-based genetic improvement programmes. In such situations, the extra selection acting on genomic information increases the challenges of obtaining unbiased VC estimates. Concerns have been raised about biased VC estimates caused by ignoring genomic information and selective genotyping [15]. Hence, it can be expected that after performing GS for several generations, the estimates of VC from a pedigree-based animal model (P-AM) would be biased due to not accounting for the impact of GS.

Single-step genomic BLUP (ssGBLUP) derived by [16–18] has provided a way to predict breeding values using phenotype, pedigree and genomic information simultaneously for both genotyped and non-genotyped individuals via a combined relationship matrix (**H**). Thus, in contrast to typical genomic BLUP (GBLUP) [19, 20] using phenotypes only from genotyped individuals, a large number of historical phenotypes from non-genotyped individuals can also be used for analyses. Alternatively, a single-step Bayesian regression model (ssBR) was proposed by [21, 22]. In contrast to ssGBLUP, in which markers for non-genotyped individuals are implicitly imputed, ssBR requires the explicit imputation of the markers for non-genotyped individuals, followed by fitting of the marker effects in the model. Previous studies have reported that ssGBLUP and the original ssBR model perform equally in terms of accuracy of prediction when assuming that VCs are known [23, 24]. However, an attractive feature of ssBR that has been neglected is that ssBR offers separate estimation of marker variance (${\sigma}_{\alpha}^2$) and total genetic variance (${\sigma}_g^2$, denoted as ${\sigma}_{\varepsilon}^2$ in ssBR). Therefore, ssBR could be employed as a variance component model for VC estimation that includes two additive genetic components.

The compatibility between pedigree-based relationships and marker-based relationships is a crucial factor for ensuring unbiased estimation when using single-step methods [19, 25, 26]. This concern is due to the different base populations to which these two relationships refer. More specifically, the pedigree-based relationship refers to a founder population in which individuals are assumed to be unrelated (denoted as P-base hereafter). The base population of marker-based relationships can be defined as the population from which the allele frequencies were obtained to compute the relationships (denoted as G-base hereafter) [27, 28]. Therefore, the VCs estimated using single-step methods with phenotypes from a population undergoing GS may not be directly comparable with the VCs estimated using pedigree-based methods [29]. The objectives of this study were (1) to examine the effects of genomic selection on estimates of VC based on the scenario of choosing phenotypes from different phases of breeding programme and (2) to investigate the estimation of VCs using different methods in a population undergoing GS.

## Results

To determine the effects of GS on the estimation of VCs, we analysed data from three scenarios reflecting no application of GS (Pheno_1_), application of GS (Pheno_1 + 2_), and a lack of information from the previous conventional breeding scheme (Pheno_2_). Table 2 presents the means and standard deviations (SDs) of the estimated VCs and heritabilities over replicates. In general, the use of single-step methods with all the phenotypes and genotypes (Pheno_1 + 2_) yielded biased estimates of the total genetic variance of P-base. Using P-AM with all the phenotypes (Pheno_1 + 2_) produced unbiased VC estimates and heritability of P-base. When the phenotypes from the conventional breeding phase were excluded (Pheno_2_), P-AM led to the underestimation of genetic variance (*P* < 0.01) of P-base. As expected, we obtained unbiased VC estimates and heritability when using P-AM with phenotypes only from the conventional phase (Pheno_1_).

For ssGBLUP, in general, the estimates of genetic variance were all biased (*P* < 0.005) across all the scenarios (Table 2). In contrast to the VCs from P-base, when using phenotypes only from the conventional phase (Pheno_1_), the genetic variance was significantly underestimated, and the residual variance was significantly overestimated. Furthermore, when including phenotypes from the GS phase in the model (Pheno_1 + 2_), the genetic variance was significantly underestimated, although unbiased estimated residual variance was observed. In contrast to the VCs from G-base, when ignoring data from the conventional breeding phase (Pheno_2_), ssGBLUP significantly overestimated genetic variance, although an unbiased estimate of residual variance was obtained.

For ssBR, the convergence of the Gibbs sampler was assessed by estimating Monte Carlo error (MCE) (via batch means). The MCEs for the estimates of the total genetic variance and residual variance were at the level of 10^− 3^, and for the estimates of marker variances, they were at the level of 10^− 6^. Two estimated genetic variances are reported (Table 2). In contrast to VCs from P-base, when using phenotypes only from the conventional phase (Pheno_1_), unbiased total genetic variance and residual variance were obtained. This led to an unbiased estimate of heritability. However, the marker-based genetic variance was significantly overestimated (*P* < 0.001) compared to the variance in G-base, resulting in a biased estimate of heritability. Conversely, when including phenotypes from the GS phase in the model (Pheno_1 + 2_), the marker-based genetic variance was unbiased, but the total genetic variance was significantly underestimated (*P* < 0.001). In contrast to the VCs from G-base, when ignoring data from the previous conventional breeding phase (Pheno_2_), ssBR yielded unbiased estimates of the total genetic variance and marker-based genetic variance, whereas the residual variance was overestimated.

## Discussion

In this study, we addressed the effects of GS on estimates of VCs. Two single-step methods (ssGBLUP and ssBR), together with the traditional pedigree-based animal model (P-AM), were used and compared based on simulated datasets that mimic dairy cattle populations. The first question addressed in this study was aimed at determining the impact of the choice of phenotypes from different phases of a breeding program on the estimation of VCs. The results showed that both the ssGBLUP and ssBR models led to biased VC estimates across all scenarios.

### Selection of data to be included in the estimation of genetic variance

We showed that P-AM yielded unbiased estimates of VC when including the phenotypes from the GS phase (Pheno_1 + 2_). We also demonstrated that when using phenotypes only from the conventional selection phase (Pheno_1_), P-AM produced unbiased VC estimates. The scenarios of Pheno_2_, which reflects current breeding programmes using GS, led to biased estimates of VC by using P-AM. This was mainly caused by ignoring the information from the selection decisions. Another possible cause of this bias might be explained by the negative LD between QTLs across the genome [30, 31]. In particular, this negative LD is stronger in a population selected according to GS information than in a population selected based on only the pedigree relationships [the mean (SD) variance of TBV for animals from generation 35 decreased to 2.46 (0.04)].

Reductions in genotyping costs have contributed to the comprehensive implementation of GS in many genetic selection programmes. This offers the opportunity to select for new traits that are difficult to measure or not yet among the breeding goals. In this situation, a new phenotyping strategy might be used, or new phenotypes may be more likely to be collected from populations under intense GS. In the present study, based on the scenario of Pheno_2_ with P-AM, our results confirmed the reduction of genetic variance due to ignoring the information from the conventional breeding phase; i.e., there are no phenotypes to account for the selection conducted in the previous period, and previous selection cannot be properly handled in the current model. Therefore, the use of P-AM including only the phenotypes from the GS phase (Pheno_2_) resulted in biased estimates of VC.

### Changed base population

With respect to the genomic relationship, an arbitrary base population may exist close to the current population undergoing GS. It can be expected that using such a base population (more recent) would lead to smaller estimates of genetic variance than using P-base. Therefore, in this study, the G-base corresponding to the current GS phase was defined separately from P-base. Consequently, it may be improper to compare VCs estimated in the GS phase (e.g., Pheno2) with the VCs in P-base, which is generally referred to by the pedigree. This concern was initially raised by Powell et al. [25]; that is, VCs estimated using genetic markers can be erroneous since an inconsistent base population is defined compared to the base population with common founders. A similar study was conducted by Veerkamp et al. [32] based on a small dataset from Holsteins, where VCs for milk yield, dry matter intake, and body weight were estimated with P-AM, GBLUP, and ssGBLUP. Their results showed that ssGBLUP produced the most precise estimates of VCs; however, they questioned whether the VCs obtained using genomic information might not be comparable with the VCs obtained using only pedigree information since these relationship matrices might refer to different base populations. Our results confirmed that when phenotypes only from the GS phase (Pheno_2_) were used, both ssGBLUP and ssBR yielded smaller genetic variance estimates compared with the VCs in P-base (Table 2).

In the present simulation study, we directly used the allele frequencies calculated from P-base to avoid the compatibility issue between the **G** and **A**_22_ matrices in ssGBLUP [26, 27, 33]. In practice, the allele frequencies of P-base are unknown, but they can be estimated using the approach proposed by Gengler et al. [34] by regressing the gene contents of ancestors on the genotypes of the progenies.

### Two estimates of genetic variance in ssBR

As originally introduced by Fernando et al. [21, 22], the ssBR model is essentially a marker effect model with all markers fitted in the model. Consequently, this feature results in a model with two estimates of additive genetic variances, i.e., the total genetic variance (${\sigma}_{\varepsilon}^2$) and the marker-based genetic variance, which can be obtained by multiplying $\sum \limits_{j=1}^m2{p}_j\left(1-{p}_j\right)$ by the estimated marker variance (${\sigma}_{\alpha}^2$). When using phenotypes only from the conventional phase (Pheno_1_), the estimated total genetic variance was unbiased; however, the marker-based genetic variance was biased upwards, although the allele frequencies from P-base were used. This result can be explained by the fact that a small proportion (1.2%) of animals (only progeny-tested bulls) in the pedigree were genotyped, resulting in poor imputation for non-genotyped animals and biased estimation of marker variance.

Apart from the estimated marker variance, the total genetic variance shows a relationship with the conditional variance of the breeding values of non-genotyped individuals (**g**_1_) given the breeding values of genotyped individuals (**g**_2_), i.e., $Var\left({\mathbf{g}}_1|{\mathbf{g}}_2\right)=\left({\mathbf{A}}_{11}-{\mathbf{A}}_{12}{\mathbf{A}}_{22}^{-1}{\mathbf{A}}_{21}\right){\sigma}_g^2$ [16]. With the assumption of multivariate normality, the markers of non-genotyped individuals were imputed via a pedigree-based linear system. A residual vector (**ε**) accounting for the remaining portion of the breeding values that could not be modelled by the imputed markers was added to the marker-based breeding values to obtain the final **g**_1_. The accuracy of this imputation quality depends on the genetic relationships between genotyped and non-genotyped animals; i.e., it is expected that less precise imputation will be achieved for old ancestors than for younger ones. More specifically, the imputation is based on a linear relationship between genotyped and non-genotyped individuals, which may not approximate the distribution of marker genotypes very well [21]. Thus, better methods (e.g., an imputation method based on a peeling algorithm [35]) for imputing the genotypes of non-genotyped individuals conditional on the genotyped individuals would be expected to yield more accurate results. In addition, as pointed out by [21], in the single-step method, we do not observe **g**_2_, but **M**_2_; this indicates that the conditioning is on the observed marker information, and the conditional genetic variance estimated in ssBR is therefore actually only an approximation of the genetic variance.

## Conclusions

This study contributes to a better understanding of the effects of GS on VC estimation. The results show that neither of the single-step models (ssGBLUP and ssBR) can precisely estimate the VCs for populations undergoing GS. Furthermore, this study has demonstrated that when the complete data are analysed with both pre-GS data and data from the GS phase, the classic P-AM can yield unbiased estimates of VC. Therefore, an implication of these findings is that the best solution for obtaining unbiased estimates of VC is to use P-AM with phenotypes from the conventional phase or phenotypes from both conventional and GS phases.

## Methods

### Simulation of data

Populations that were similar to the Danish Jersey dairy cattle population in terms of the breeding scheme and population structure were simulated over a 35-year period with 5 replicates for each scenario. The simulation was conducted with the following three phases: (1) The historical phase, covering 3000 non-overlapping generations, was run to generate an initial linkage disequilibrium (LD) structure. The simulated genome consisted of 30 chromosomes of 100 centiMorgans (cM) each with 100 QTLs and 10,000 biallelic SNP markers. The QTLs and markers were uniformly distributed within each chromosome. This resulted in a total of 3000 QTLs and 300,000 markers across the whole genome. The offspring inherited alleles at these loci from their parents following Mendel’s rules allowing for mutation (assumed only to happen in the historical phase) and recombination. A recurrent mutation rate of 2.5 × 10^− 5^ for both markers and QTLs was set to establish mutation-drift equilibrium in the historical generations. The number of recombination per chromosome (per Morgan) was sampled from a Poisson distribution with a mean equal to the length of the chromosome, and crossovers were uniformly located along the chromosome. This part of the simulation was implemented with QMSim software [36]. Generation 3000 was used as the base population, in which 40,000 SNPs were randomly chosen from the pool of 300,000 markers, and 2000 QTLs were randomly chosen from the pool of 3000 QTLs. (2) In the next phase, 20 years of conventional breeding were simulated. Each year, 50 young bulls were selected based on their parent average (PA) and the progeny tested, and 10,000 cows in different age groups were maintained. Only cows in the first lactation, however, were assumed to have phenotypes. At the end of phase (2), all proven bulls were genotyped. (3) In the last phase, 15 years of genomic selection were simulated. Each year, 500 bulls and 2000 heifers were selected for genotyping at one year of age based on their PA genomic estimated breeding values (GEBV) computed by ssGBLUP. After genomic evaluation, 50 of these 500 bulls were selected for breeding. The simulations of phase (2) and (3) were performed with ADAM software [37]. For the herd-year-season (HYS) effect (contemporary group effect), individuals were allocated to 100 herds, 35 years and 4 seasons.

### Scenarios

Three scenarios based on the use of phenotypic information from different phases of breeding programs for the estimation of VCs were explored: (1) Pheno_1_: phenotypes only from conventional phase (1–20 years) were used; (2) Pheno_1 + 2_: phenotypes from both the conventional phase and genomic selection phase (1–35 years) were used; (3) Pheno_2_: phenotypes from only the genomic selection phase were used (21–35 years). Within each scenario, the three single-trait models (P-AM, ssGBLUP, and ssBR) were applied. An overview of the subsets used and the average number of individuals in the pedigree, phenotypes, and genotypes for each scenario over 5 replicates are shown in Table 1. For validation, VCs and heritabilities from two different base populations (P-base and G-base) are presented in Table 2. The genetic variance of P-base was calculated from the variance of “true” breeding values (TBVs) based on animals from the founder population, while the genetic variance of G-base was calculated from the variance of TBVs based on animals from years 18, 19, and 20, i.e., the last three years before the start of GS (animals in G-base were related). Hence, when genomic information was included and phenotypes were only collected from GS phase (Pheno_2_), the VC estimates needed to be compared with the VCs from G-base.

**Table 1:** Scenarios based on choosing different phases of phenotypes for variance component estimation and average statistics of simulated datasets over 5 replicates

	Phenotyping periods (years)				No. of genotypes		
Scenario^a^	Conventional phase (1–20)	Genomic selection phase (21–35)	Size of pedigree	No. of phenotypes	Bulls	Cows with phenotype	Cows without phenotype
Pheno_1_	√	–	84,164	81,240	1050	–	–
Pheno_1 + 2_	√	√	160,131	144,728	8550	25,021	4979
Pheno_2_	–	√	106,011	63,487	8550	25,021	4979

^a^Pheno_1_: phenotypes from only the conventional phase (1–20 years) were used; Pheno_1 + 2_: phenotypes

from both the conventional phase and genomic selection phase (1–35 years) were used; Pheno_2_:

phenotypes from only the genomic selection phase were used (21–35 years)

**Table 2:** Mean (SD) of the “true” variance components and heritability in the base (founder) population (P-base) and the base population for the genomic phase^a^ and estimates of variance components and heritabilities from P-AM, ssGBLUP, and ssBR based on three scenarios of phenotyping^b^

Method	Scenario^b^	${\sigma}_g^2$(${\sigma}_{\varepsilon}^2$)	${\sigma}_e^2$	${\sigma}_{\alpha}^2\sum \limits_{j=1}^m2{p}_j\left(1-{p}_j\right)$	*h*^*2c*^
True VCs in P-base		3.59 (0.03)	5.05 (0.09)	–	0.42 (0.004)
True VCs in G-base		2.82 (0.09)	5.10 (0.07)	–	0.36 (0.009)
P-AM	Pheno_1_	3.62 (0.12)	5.02 (0.02)	–	0.42 (0.009)
	Pheno_1 + 2_	3.52 (0.06)	5.07 (0.03)	–	0.41 (0.004)
	Pheno_2_	3.19 (0.20)^*^	5.21 (0.10)	–	0.38 (0.018)^*^
ssGBLUP	Pheno_1_	2.99 (0.12)^***^	5.54 (0.01)^***^	–	0.35 (0.009)^***^
	Pheno_1 + 2_	3.39 (0.06)^***^	5.17 (0.03)	–	0.40 (0.009)^***^
	Pheno_2_	3.36 (0.15)^***^	5.19 (0.05)	–	0.39 (0.013)^*^
ssBR	Pheno_1_	3.44 (0.15)	5.10 (0.04)	4.20 (0.10)^***^	0.40 (0.012)
	Pheno_1 + 2_	3.14 (0.10)^***^	5.24 (0.02)^*^	3.71 (0.11)	0.37 (0.008)^***^
	Pheno_2_	2.99 (0.20)	5.27 (0.06)^**^	2.97 (0.09)	0.36 (0.017)

^a^P-AM: traditional pedigree-based animal model; ssGBLUP: single-step genomic BLUP; ssBR: single-step Bayesian regression. ${\sigma}_g^2$ is the genetic variance used in P-AM, ${\sigma}_{\varepsilon}^2$ is the total genetic variance used in ssBR; ${\sigma}_e^2$ is the residual variance; ${\sigma}_{\alpha}^2$ is the marker variance; ${\sigma}_{\alpha}^2\sum \limits_{j=1}^m2{p}_j\left(1-{p}_j\right)$ is used to calculate genetic variance via the estimated marker variance in ssBR, where *p*_*j*_ is the observed allele frequency at locus *j*, and m is the total number of markers

^b^Pheno_1_: phenotypes from only the conventional phase (1–20 years) were used; Pheno_1 + 2_: phenotypes from both the conventional phase and genomic selection phase (1–35 years) were used; Pheno_2_: phenotypes from only the genomic selection phase were used (21–35 years)

^c^Heritabilities (*h*^2^) from P-AM, ssGBLUP, and ssBR calculated as $\frac{\sigma_{g\left(\varepsilon \right)}^2}{\left({\sigma}_{g\left(\varepsilon \right)}^2+{\sigma}_e^2\right)}$

The significance test was performed to determine whether the estimated parameter differs from the simulated parameter. ^*^ significant at *P* < 0.01; ^**^ significant at *P* < 0.005; ^***^ significant at *P* < 0.001

### Statistical models for VC estimation

#### P-AM

The classical animal model [38] using pedigree-based relationships can be written as follows:

1 $$ \mathbf{y}=\mathbf{X}\boldsymbol{\upbeta } +\mathbf{Za}+\mathbf{e} $$

where **y** represents the vector of phenotypes; **β** is a vector of fixed effects, i.e., herd-year-season effects: **a** is a vector of additive genetic effects following $N\left(\mathbf{0},\mathbf{A}{\sigma}_g^2\right)$, where **A** is the numerator relationship matrix, and ${\sigma}_g^2$ is the additive genetic variance; **X** and **Z** are design matrices relating phenotypes to fixed effects and random animal effects, respectively; and **e** is a vector of residuals following $N\left(\mathbf{0},\mathbf{I}{\sigma}_e^2\right)$, where ${\sigma}_e^2$ is the residual variance.

#### ssGBLUP

The model equation of the regular ssGBLUP model [16, 17] was the same as model (1) but used an **H** matrix that combines the marker-based (**G**) and pedigree-based (**A**) relationship matrices to replace the numerator relationship matrix (**A)** in the classical animal model. Therefore, the vector of GEBVs is assumed to be distributed as $N\left(\mathbf{0},\mathbf{H}{\sigma}_g^2\right)$. The mixed model equations (MME) require the inverse of the **H** matrix [18]:

2 $$ {\mathbf{H}}^{-1}={\mathbf{A}}^{-1}+\left[\begin{array}{cc}\mathbf{0}& \mathbf{0}\\ {}\mathbf{0}& {\mathbf{G}}^{-1}-{\mathbf{A}}_{22}^{-1}\end{array}\right] $$

where ${\mathbf{A}}_{22}^{-1}$ is the inverse of the pedigree-based relationship matrix for the genotyped individuals, and **G** is constructed according to [19]:

3 $$ \mathbf{G}=\frac{\mathbf{Z}{\mathbf{Z}}^{\prime }}{\sum_{j=1}^m2{p}_j\left(1-{p}_j\right)} $$

where **Z** is a centred marker covariate matrix containing 0 − 2*p*_*j*_, 1 − 2*p*_*j*_, and 2 − 2*p*_*j*_ for genotypes of AA, AB, and BB, respectively; *p*_*j*_ is the allele frequency at locus *j*, and m is the total number of markers. For the scenarios of Pheno_1_ and Pheno_1 + 2_, the allele frequencies were estimated from the animals in P-base, whereas for the scenario of Pheno_2_, the allele frequencies were estimated from the animals in G-base. The variance components of P-AM and ssGBLUP were estimated with REML [2] using the average information algorithm (AIREML) [8] as implemented in the DMU package [39].

#### ssBR

An ssBR model [21] based on BayesC [40, 41] with the assumption of all markers having non-zero effects (i.e., π = 0) and a common variance for all markers can be specified as follows:

4 $$ \left[\begin{array}{c}{\mathbf{y}}_1\\ {}{\mathbf{y}}_2\end{array}\right]=\left[\begin{array}{c}{\mathbf{X}}_1\\ {}{\mathbf{X}}_2\end{array}\right]\boldsymbol{\upbeta} +\left[\begin{array}{cc}{\mathbf{Z}}_1& \mathbf{0}\\ {}\mathbf{0}& {\mathbf{Z}}_2\end{array}\right]\left[\begin{array}{l}{\mathbf{M}}_{\mathbf{1}}\boldsymbol{\upalpha} +\boldsymbol{\upvarepsilon} \\ {}{\mathbf{M}}_{\mathbf{2}}\boldsymbol{\upalpha} \end{array}\right]+\mathbf{e} $$

where subscript 1 denotes non-genotyped individuals, and subscript 2 denotes genotyped individuals. Thus, **y** represents the vector of phenotypes; **β** is a vector including elements of herd-year-season effects; **X** and **Z** are design matrices; **M**_2_ contains observed marker covariates for genotyped individuals; **M**_1_ contains imputed marker covariates for non-genotyped individuals; the imputation is conducted from the following linear relationship: ${\mathbf{M}}_1={\mathbf{A}}_{12}{\mathbf{A}}_{22}^{-1}{\mathbf{M}}_2$ based on the assumption of multivariate normality, where **A**_12_ and **A**_22_ are the sub-matrices of **A**; **α** is the vector of marker effects $\boldsymbol{\upalpha} \sim N\left(\mathbf{0},\mathbf{I}{\sigma}_{\alpha}^2\right)$; **ε** is the vector of imputation residual deviations, $\boldsymbol{\upvarepsilon} \sim N\left(\mathbf{0},\left({\mathbf{A}}_{11}-{\mathbf{A}}_{12}{\mathbf{A}}_{22}^{-1}{\mathbf{A}}_{21}\right){\sigma}_{\upvarepsilon}^2\right)$, due to the inaccuracy of imputation, where **A**_11_, **A**_12_, **A**_22_ and **A**_21_ are the sub-matrices of **A**; ${\sigma}_{\alpha}^2$ is the variance of the marker effects under the assumption that all markers exhibit common genetic variance and can explain all additive genetic variance; ${\sigma}_{\varepsilon}^2$ is the imputation residual variance; and **e** is a vector of residuals, $\mathbf{e}\sim N\left(\mathbf{0},\mathbf{I}{\sigma}_e^2\right)$. For location parameters, a flat prior is assigned for **β**, and normal priors are specified for **α**, **ε**, and **e**, with null mean and variance of ${\sigma}_{\alpha}^2$, ${\sigma}_{\varepsilon}^2$, and ${\sigma}_e^2$, respectively. For dispersion parameters, scaled inverse chi-squared distributions with scale factors, ${S}_{\alpha}^2=0.0002$, ${S}_{\varepsilon}^2=2$, ${S}_e^2=2.5$, and degree of freedom, *ν*_*α*(ε, *e*)_ = 4, are assumed to be the prior distributions for ${\sigma}_{\alpha}^2$, ${\sigma}_{\upvarepsilon}^2$, and ${\sigma}_e^2$, respectively. The final GEBV for individuals can be written as follows:

5 $$ \mathbf{g}=\left[\begin{array}{c}{\mathbf{g}}_1\\ {}{\mathbf{g}}_2\end{array}\right]=\left[\begin{array}{l}{\mathbf{M}}_{\mathbf{1}}\boldsymbol{\upalpha} +\boldsymbol{\upvarepsilon} \\ {}{\mathbf{M}}_{\mathbf{2}}\boldsymbol{\upalpha} \end{array}\right] $$

where **g**_1_ and **g**_2_ represent the vectors of GEBV for non-genotyped and genotyped individuals, respectively.

The use of ssBR allows the inference of the additive genetic variance from two sources of information: first, the total genetic variance, approximated by the estimated imputation residual variance, ${\sigma}_{\varepsilon}^2$; second, the marker-based genetic variance, computed as the estimated marker variance, ${\sigma}_{\alpha}^2$, multiplied by $\sum \limits_{j=1}^m2{p}_j\left(1-{p}_j\right)$, where *p*_*j*_ is the allele frequency at locus *j* and m is the total number of markers. Similar to ssGBLUP, for the scenarios of Pheno_1_ and Pheno_1 + 2_, allele frequencies were calculated based on the stored genotypes in the base population (P-base). For the of Pheno_2_ scenario, the allele frequencies were calculated based on the current genotyped population. In addition, an extra fixed effect (*μ*_*g*_) was fitted in the model to account for the unknown expectation for genotyped individuals [21]. The ssBR program was written in Fortran 95. A Gibbs sampler was used to draw inferences for all model parameters from their posterior distributions. The length of the chain was set to 50,000, with a burn-in of 20,000 iterations. The convergence of the posterior distribution for each parameter investigated was assessed using the boa and coda packages [42, 43].

## Data Availability

The datasets analysed during the current study are available in the figshare repository (10.6084/m9.figshare.10547921.v3).

## Abbreviations

cM: Centimorgan
GEBV: Genomic estimated breeding values
GS: Genomic selection
HYS: Herd-year-season
MCMC: Markov chain Monte-Carlo
PA: Parent average
P-AM: Pedigree-based animal model
REML: Restricted maximum likelihood
ssBR: Single-step Bayesian regression
ssGBLUP: Single-step genomic BLUP
VC: Variance components

## References

[1] Hofer A (1998). Variance component estimation in animal breeding: a review. J Anim Breed Genet.

[2] Patterson HD, Thompson R (1971). Recovery of inter-block information when block sizes are unequal. Biometrika..

[3] Meyer K (1990). Present status of knowledge about statistical procedures and algorithms to estimate variance and covariance components, 4th world Congr.

[4] Smith SP, Graser HU (1986). Estimating variance-components in a class of mixed models by restricted maximum-likelihood. J Dairy Sci.

[5] Gilmour AR, Thompson R, Cullis BR (1995). Average information REML: an efficient algorithm for variance parameter estimation in linear mixed models. Biometrics..

[6] Johnson DL, Thompson R (1995). Restricted maximum-likelihood-estimation of variance-components for Univariate animal-models using sparse-matrix techniques and average information. J Dairy Sci.

[7] Madsen P, Jensen J, Thompson R (1994). Estimation of (co)variance components by REML in multivariate mixed linear models using average of observed and expected information, 5th world Congr.

[8] Jensen J, Mäntysaari EA, Madsen P, Thompson R (1997). Residual maximum likelihood estimation of (co) variance components in multivariate mixed linear models using average information. J Indian Soc Agric Stat.

[9] Ducrocq V (1990). Estimation of genetic parameters arising in nonlinear models, 4th world Congr.

[10] Gianola D, Fernando RL (1986). Bayesian methods in animal breeding theory. J Anim Sci.

[11] Gianola D, Foulley JL (1990). Variance-estimation from integrated likelihoods (veil). Genet Sel Evol.

[12] Gianola D, Foulley J, Fernando R (1986). Prediction of breeding values when variances are not known. Genet Sel Evol.

[13] Sorensen DA, Kennedy BW (1984). Estimation of genetic variances from unselected and selected populations. J Anim Sci.

[14] Martinez V, Bunger L, Hill WG (2000). Analysis of response to 20 generations of selection for body composition in mice: fit to infinitesimal model assumptions. Genet Sel Evol.

[15] Jensen J. (2016). Estimation of genetic variance in the age of genomics. Journal of Animal Breeding and Genetics.

[16] Legarra A, Aguilar I, Misztal I (2009). A relationship matrix including full pedigree and genomic information. J Dairy Sci.

[17] Christensen OF, Lund MS (2010). Genomic prediction when some animals are not genotyped. Genet Sel Evol.

[18] Aguilar I, Misztal I, Johnson DL, Legarra A, Tsuruta S, Lawlor TJ (2010). Hot topic: a unified approach to utilize phenotypic, full pedigree, and genomic information for genetic evaluation of Holstein final score. J Dairy Sci.

[19] VanRaden PM (2008). Efficient methods to compute genomic predictions. J Dairy Sci.

[20] Hayes BJ, Visscher PM, Goddard ME (2009). Increased accuracy of artificial selection by using the realized relationship matrix. Genet Res.

[21] Fernando RL, Dekkers JCM, Garrick DJ (2014). A class of Bayesian methods to combine large numbers of genotyped and non-genotyped animals for whole-genome analyses. Genet Sel Evol.

[22] Fernando RL, Cheng H, Golden BL, Garrick DJ (2016). Computational strategies for alternative single-step Bayesian regression models with large numbers of genotyped and non-genotyped animals. Genet Sel Evol.

[23] Gao H, Koivula M, Jensen J, Stranden I, Madsen P, Pitkanen T, Aamand GP, Mantysaari EA (2018). Short communication: genomic prediction using different single-step methods in the Finnish red dairy cattle population. J Dairy Sci.

[24] Lee J, Cheng H, Garrick D, Golden B, Dekkers J, Park K, Lee D, Fernando R (2017). Comparison of alternative approaches to single-trait genomic prediction using genotyped and non-genotyped Hanwoo beef cattle. Genet Sel Evol.

[25] Powell JE, Visscher PM, Goddard ME (2010). Reconciling the analysis of IBD and IBS in complex trait studies. Nat Rev Genet.

[26] Vitezica Z, Aguilar I, Misztal I, Legarra A (2011). Bias in genomic predictions for populations under selection. Genet Res.

[27] Christensen OF, Madsen P, Nielsen B, Ostersen T, Su G (2012). Single-step methods for genomic evaluation in pigs. Animal..

[28] Legarra A, Christensen OF, Vitezica ZG, Aguilar I, Misztal I (2015). Ancestral Relationships Using Metafounders: Finite Ancestral Populations and Across Population Relationships. Genetics.

[29] Legarra A (2016). Comparing estimates of genetic variance across different relationship models. Theor Popul Biol.

[30] Sorensen D, Fernando R, Gianola D (2001). Inferring the trajectory of genetic variance in the course of artificial selection. Genet Res.

[31] Lehermeier C, de los Campos G, Wimmer V, Schon CC (2017). Genomic variance estimates: With or without disequilibrium covariances?. J Anim Breed Genet.

[32] Veerkamp RF, Mulder HA, Thompson R, Calus MPL (2011). Genomic and pedigree-based genetic parameters for scarcely recorded traits when some animals are genotyped. J Dairy Sci.

[33] Christensen OF. Compatibility of pedigree-based and marker-based relationship matrices for single-step genetic evaluation. Genet Sel Evol. 2012;44:37. 10.1186/1297-9686-44-37 PMC3549765 23206367

[34] Gengler N, Mayeres P, Szydlowski M (2007). A simple method to approximate gene content in large pedigree populations: application to the myostatin gene in dual-purpose Belgian blue cattle. Animal..

[35] Meuwissen THE, Svendsen M, Solberg T, Odegard J. Genomic predictions based on animal models using genotype imputation on a national scale in Norwegian Red cattle. Genet Sel Evol. 2015;47:79. 10.1186/s12711-015-0159-8 PMC4605129 26464226

[36] Sargolzaei M, Schenkel FS (2009). QMSim: a large-scale genome simulator for livestock. Bioinformatics..

[37] Pedersen LD, Sorensen AC, Henryon M, Ansari-Mahyari S, Berg P (2009). ADAM: a computer program to simulate selective breeding schemes for animals. Livest Sci.

[38] Henderson CR (1984). Applications of linear models in animal breeding, University of Guelph, [Guelph, Ont.].

[39] Madsen P, Jensen J (2013). A User's Guide to DMU - A Package for Analysing Multivariate Mixed Models.

[40] Habier D, Fernando RL, Kizilkaya K, Garrick DJ (2011). Extension of the bayesian alphabet for genomic selection. BMC Bioinformatics.

[41] Kizilkaya K, Fernando RL, Garrick DJ (2010). Genomic prediction of simulated multibreed and purebred performance using observed fifty thousand single nucleotide polymorphism genotypes. J Anim Sci.

[42] Smith BJ (2007). boa: An R package for MCMC output convergence assessment and posterior inference. J Stat Softw.

[43] Plummer M, Best N, Cowles K, Vines K. CODA: convergence diagnosis and output analysis for MCMC. R News. 2006;6:7–11.

